# Promoting the Psychological Well-Being of Healthcare Providers Facing the Burden of Adverse Events: A Systematic Review of Second Victim Support Resources

**DOI:** 10.3390/ijerph18105080

**Published:** 2021-05-11

**Authors:** Isolde Martina Busch, Francesca Moretti, Irene Campagna, Roberto Benoni, Stefano Tardivo, Albert W. Wu, Michela Rimondini

**Affiliations:** 1 Department of Neuroscience, Biomedicine and Movement Sciences, University of Verona, Policlinico G.B. Rossi Piazzale L.A. Scuro 10, 37134 Verona, Italy; isoldemartina.busch@univr.it (I.M.B.); francesca.moretti76@gmail.com (F.M.); 2 Department of Diagnostics and Public Health, University of Verona, 37134 Verona, Italy; irene.campagna87@gmail.com (I.C.); roberto.benoni90@gmail.com (R.B.); stefano.tardivo@univr.it (S.T.); 3 Department of Health Policy and Management, Johns Hopkins Bloomberg School of Public Health, Baltimore, MD 21205, USA; awu@jhu.edu

**Keywords:** second victim, healthcare providers, support programs, peer support, emotional distress, mental health, resilience, adverse event

## Abstract

Given the negative impact of adverse events on the wellbeing of healthcare providers, easy access to psychological support is crucial. We aimed to describe the types of support resources available in healthcare organizations, their benefits for second victims, peer supporters’ experiences, and implementation challenges. We also explored how these resources incorporate aspects of Safety I and Safety II. We searched six databases up to 19 December 2019 and additional literature, including weekly search alerts until 21 January 2021. Two reviewers independently performed all methodological steps (search, selection, quality assessment, data extraction, formal narrative synthesis). The 16 included studies described 12 second victim support resources, implemented between 2006 and 2017. Preliminary data indicated beneficial effects not only for the affected staff but also for the peer responders who considered their role to be challenging but gratifying. Challenges during program implementation included persistent blame culture, limited awareness of program availability, and lack of financial resources. Common goals of the support programs (e.g., fostering coping strategies, promoting individual resilience) are consistent with Safety II and may promote system resilience. Investing in second victim support structures should be a top priority for healthcare institutions adopting a systemic approach to safety and striving for just culture.

## 1. Introduction

Complex organizations, including healthcare institutions, must deal with both the inevitability of error and the need to ensure safe and high-quality care. Avoiding mistakes that lead to adverse events and patient harm is the main aim of a high reliable organization [1]. It is well recognized how system failures at the blunt end may trigger unsafe cascades of events that affect healthcare providers’ performance and cause active failures at the sharp end. However, a blame culture persists in many organizations and unexpected patient harm is often attributed to individual workers [2,3]. When adverse events occur, the negative consequences can reach far beyond the patient. While the patient is recognized as the “first victim”, healthcare providers may also be greatly affected by a range of psychological and psychosomatic symptoms [4]. Consequences can be serious for workers’ personal and professional wellbeing, including job turnover, symptoms of post-traumatic stress disorder (PTSD), and even suicide [4,5,6,7].

Given the potential for a dramatic impact, Albert Wu coined the term “second victim”, referring to a healthcare provider harmed by an adverse event [8]. Some have subsequently criticized the use of this term, arguing that it denies any accountability on the part of healthcare providers and that it may be offensive to affected patients and families [9]. However, it is now widely understood that medical errors and patient harm are caused by multiple factors that reside in the system, and not with individual workers. These individuals are vulnerable to being harmed by the same factors that injure patients. In addition, symptoms such as remorse, guilt, shame, anxiety, and depression are highly prevalent among healthcare workers when patients are harmed by care. This suggests that attention needs to be focused on workers’ pervasive feelings of guilt and self-punishment rather than on a lack of responsibility [4,10].

These symptoms are common among second victims. Seys and colleagues found that nearly half of all healthcare providers may suffer from the emotional sequelae of an adverse event [11], whereas other studies found even higher percentages [4]. Moreover, second victims unable to effectively deal with the distress of an adverse event may also have significant consequences on their job performance. Inappropriate, maladaptive and/or dysfunctional coping strategies may further affect the wellbeing of the patient (e.g., ineffective mitigation efforts after the adverse event, ineffective communication), the healthcare provider (e.g., long-lasting impact, impact on the ability to provide safe care), and the system (e.g., defensive medicine, inability to develop a learning environment) [10,12].

This body of evidence has led to recognition of psychological support for second victims as a priority. The goal of a support program is first to ensure providers with a swift and efficient recovery from the event. One of the main principles of patient safety is to limit the reoccurrence of mistakes. A well-supported healthcare provider will be better able to move on rapidly after a stressful event, effectively support patients and caregivers affected by the adverse event, and help to identify corrective actions to prevent the reoccurrence of the same failure. Accordingly, ensuring an adequate support to second victims is recognized as an essential safety standard and strategy by most important national and international organizations (e.g., Strategy 4.4 of the Global Patient Safety Action Plan 2021-2030 of the WHO mentions, “*Ensure that patients, families and health care staff (the “second victims”) are given ongoing psychological and other support in the aftermath of a serious patient safety incident*” [13], p. 39).

While the main aim of a safety program (also called Safety I) is to limit the occurrence of harmful events and their consequences at the patient, healthcare provider and system level, recent literature has focused on another aspect of safety, namely Safety II. In this approach, resilience is a key element to ensure the delivery of safe care. Hollnagel and colleagues, who first underlined the importance of this approach, state that “*We should acknowledge that things go right because clinicians are able to adjust their work to conditions*” and that “*acceptable outcomes and adverse outcomes have a common basis, namely everyday performance adjustment*” [14], p. 20. This vision identifies healthcare providers as valuable resources for ensuring safe care, rather than as a source of inevitable mistakes due to their human fallibility.

Safety management programs should integrate their ability to learn from “what goes wrong” (i.e., Safety I) and the ability to acknowledge what happens when “things go right” (i.e., Safety II). A corollary of this approach is that a second victim support program should aim not only to help healthcare providers to rapidly recover and limit the negative consequences involvement in an adverse event, but also to develop new resources to promote resilience. 

But how are such support programs actually implemented in healthcare settings worldwide? On what concepts are they based? What is their impact on stakeholders and their effectiveness? 

There have been few reviews on this topic. For instance, a literature review by Stone [15] offered a relatively short overview of second victim support programs implemented in the past decade without applying a rigorous methodological approach, and the scoping review by Wade et al. [16] adopted primarily an organizational viewpoint on second victim support in acute care settings. 

However, a systematic review following a strict methodology, including the preparation and registration of the review protocol as well as risk of bias assessment [17], was still missing. Therefore, the aim of this study was to conduct a systematic, in-depth description and analysis of the type of second victim support programs available in healthcare organizations, their benefits for second victims, peer supporters’ experiences, and challenges encountered during the implementation of the programs. Further, our systematic review sought to describe the conceptual basis of the programs and explore in which ways the programs incorporate aspects of the Safety I and Safety II approach. 

## 2. Materials and Methods

The present study is registered at PROSPERO—International prospective register of systematic reviews (Record ID: CRD42020157488). 

### 2.1. Searches

We conducted a systematic search of six electronic databases (i.e., PubMed, Web of Science, Scopus, PsycINFO, MEDLINE, ScienceDirect) up to 18 December 2019, and used the following search strategy: (medical error OR patient safety incident OR near miss OR second victim) AND (health professional OR health care provider) AND (support program OR support strategy OR support protocol OR support system) (see Supplementary File S1). There were no restrictions to publication date and language. To detect additional literature, we screened grey literature databases, volumes of journals, reference lists of books, consensus statements, white papers, reviews, and websites dedicated to second victims. Furthermore, to identify newly published articles, we created automatic, weekly search alerts for the databases PubMed and Web of Science from 18 December 2019 through 21 January 2021 (see Supplementary File S2). Two reviewers (R.B. and I.C.) independently screened record titles and abstracts with the Systematic Reviews Web application Rayyan [18] and assessed the full texts that were considered potentially eligible by at least one of the two reviewers. A third reviewer (I.M.B.) was involved in case of dissent. 

We followed the Reporting Items for Systematic Reviews and Meta-Analyses (PRISMA) guidelines by Moher et al. [19] and provide, as recommended by Higgins and Deeks [20], a list of the characteristics of the excluded studies. 

### 2.2. Inclusion and Exclusion Criteria

We considered studies eligible for inclusion if

(1) the development and/or the implementation and/or the evaluation of support resources for second victims (i.e., support program, toolkit, course) were described. Second victims include all healthcare providers (e.g., physicians, nurses, midwives) involved in adverse events/patient safety incidents regardless of their profession, age or other sociodemographic characteristics;
(2) the support program was described in detail elucidating every step of the support strategy;
(3) the support program was part of a structured intervention organized and/or promoted by their healthcare institution.

We excluded editorials, letters, and reviews of all types (e.g., scoping reviews, narrative reviews, systematic reviews). 

### 2.3. Risk of Bias Assessment

Two independent raters (I.M.B., F.M.) assessed the quality of the included studies using the *Joanna Briggs Institute Critical Appraisal Checklist for Text and Opinion Papers* [21] and *the Mixed Methods Appraisal Tool* (MMAT) (Version 18) for qualitative studies, quantitative nonrandomized studies, and quantitative descriptive studies [22]. Since the included studies differed in study design, we selected the tools accordingly. Any potential dissent was discussed, including a third rater (M.R.) to adjudicate. 

### 2.4. Outcome Measures 

The main outcomes were

(1) a descriptive overview of second victims support resources developed around the world;
(2) their conceptual basis, including aspects/elements of the Safety I (i.e., mostly reactive approach focusing on identifying risks and causes of adverse outcomes, and limiting their reoccurrence) and Safety II approach (i.e., proactive approach focusing on identifying resources and key elements of a positive performance, and promoting resilience at the individual and institutional level);
(3) programs’ benefits for second victims;
(4) personal perceptions and experiences of peer supporters; and
(5) challenges encountered during the implementation of the support resources.

### 2.5. Data Extraction and Synthesis

Two reviewers (R.B., I.C.) independently extracted characteristics and outcome measures of the selected studies using a data collection form in Microsoft Excel. Disagreements were discussed and, where necessary, a third reviewer (I.M.B.) was involved. If missing data were detected, the study authors were contacted.

We conducted a formal narrative synthesis of the extracted results and present in a narrative text the above-mentioned outcome measures as well as a structured tabulation of second victim support resources that were described, a timeline of their implementation, and a figure illustrating the link between individual and system resilience. 

To homogenize data presentation and improve data synthesis, we performed in some cases small calculations or conversions (e.g., conversion of absolute frequencies into percentages) (see footnotes of Supplementary File S4). 

If studies, which were published in different years but focusing on the same program, reported data on the same variables (e.g., number of peer support encounters), we extracted the data from the most recent publication. 

## 3. Results

**Figure 1:**
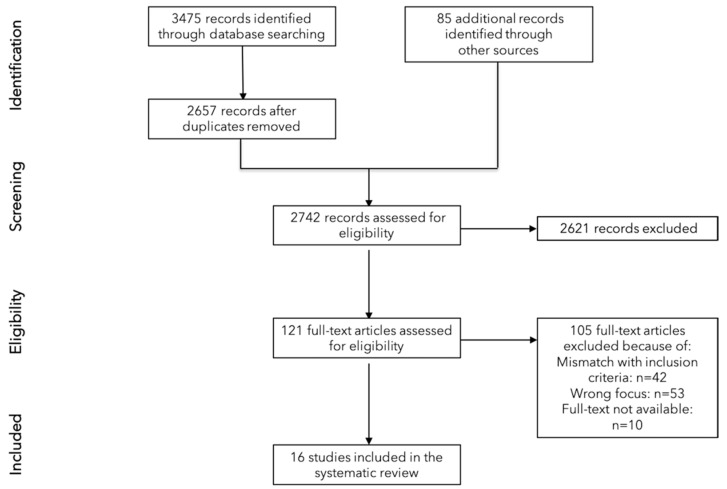
PRISMA Flow Diagram.

We retrieved 2657 records from the databases and identified 85 additional records through other sources. After screening these 2742 records for eligibility, we assessed 121 full-text articles of which 105 were excluded due to various reasons (e.g., mismatch with the inclusion criteria, wrong focus) (see Supplementary File S3) and included a final number of 16 articles [23,24,25,26,27,28,29,30,31,32,33,34,35,36,37,38] (see Figure 1). 

We contacted the authors of the studies in three cases to ask for additional information, but only two author groups responded. The primary studies, all written in English, were published between 2008 [37] and 2021 [23]. Aside from two papers from Spain [30] and Indonesia [37], all other studies were conducted in the United States [23,24,25,26,27,28,29,30,32,33,34,35,36,37]. There were quantitative nonrandomized studies [38], quantitative descriptive studies [27,28,29,30,31], mixed-method studies [23,24,25,26,35], qualitative studies [36] and text and opinion papers [32,33,34,37]. 

### 3.1. Risk of Bias Assessment 

All but one study [29] assessed with the MMAT Tool met four of the five quality criteria, with seven studies fulfilling all [23,24,25,27,35,36]. As regards the JBI Critical Appraisal Checklist for Text and Opinion Papers, all four articles met more than half of the six quality criteria [32,33,34,37], with three meeting all [32,33,37] (see Table 1). 

### 3.2. Second Victim Support Resources 

#### 3.2.1. Main Characteristics 

The 16 included studies described 10 second victim programs and two other support resources (i.e., Medically Induced Trauma Support Services (MITSS) Toolkit for building a Clinician and Staff Support Program [33]; Mitigating Impact in Second Victims (MISE) website and online training course [31]). 

Several studies focused on the same programs [23,24,25,27,28,30,35]. Supplementary File S4 gives a detailed overview of the different support resources and outcomes reported by the primary studies.

**Figure 2:**
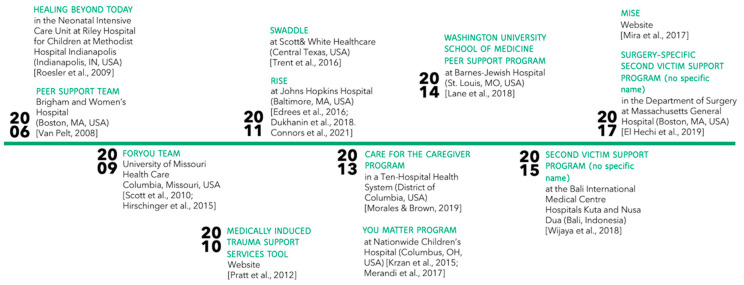
Implementation of second victim support resources.

The first second victim support resources were implemented in 2006 (i.e., Healing beyond Today, Peer Support Service at Brigham and Women’s Hospital), the most recent in 2017 (i.e., MISE [31], Surgery-Specific Second Victim Support Program [26]). Figure 2 presents a detailed timeline of the implementation of the support structures. 

The programs differed slightly in the applied terminology, using terms such as “adverse events” (Peer Support Service), “stressful clinical event” (forYOU Team), “stressful patient-related events” (RISE), “serious, unanticipated adverse events” (Care for the Caregiver Program), “medical errors and adverse patient outcomes” (YouMatter Program), “medical errors and adverse events” (WUSM Peer Support Program), and “major perioperative adverse events” (Surgery-Specific Second Victim Support Program). 

Three programs extended their focus over time, including workplace violence incidents (forYOU Team and RISE) [39], burnout, grief, and domestic abuse (Care of the Caregiver Program). 

Aside from Healing Beyond Today, support was usually provided by internal peers. Generally, the support was voluntary, confidential, and available immediately or shortly after the event, and allowed referrals to higher levels of support, if necessary. While the majority of programs offered both one-on-one and group support (Peer Support Service, Healing Beyond Today, forYOU Team, RISE, YOU Matter Program, Second Victim Support Program at the Bali International Medical Centre Hospitals), four programs provided only one-on-one support (Swaddle, Care for the Caregiver Program, WUSM Peer support program, Surgery-specific second victim support program). To identify affected staff, programs mostly relied on self-identification and identification by colleagues/peers. Three programs (Surgery-Specific Second Victim Support Program, Healing Beyond Today, WUSM Peer Support Program) also applied more proactive methods (e.g., identification of adverse events and involved providers). 

**Figure 3:**
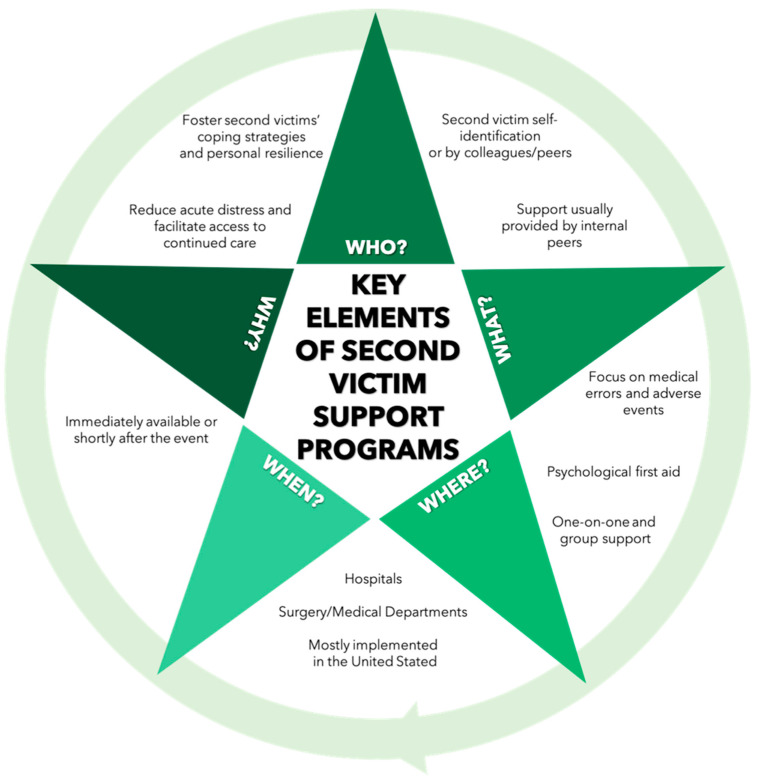
Key elements of second victim support programs.

While most of the studies [25,26,28,29,32,35,36,37] noted that training sessions were required to become a peer supporter, only a few studies, referring to RISE and the forYOU Team [25,27], mentioned regular meetings and ongoing training of peer supporters as well as debriefings after the encounters. Figure 3 presents an overview of the key elements of second victim support programs. 

#### 3.2.2. Conceptual Basis of Second Victim Support Resources

Most of the support resources used an integrated approach, including different concepts and perspectives. 

The Medically Induced Trauma Support Services (MITTS) founded by Kenney in 2002 [37] and the MITTS toolkit developed by Pratt et al. [33] served as a theoretical basis for the forYOU Team, Swaddle, and RISE. Psychological approaches for immediate crisis intervention, namely Critical Incident Stress Management [40,41] and Psychological First Aid, acted as guidance for many programs, such as Peer Support Service, Healing Beyond Today, Care for the Caregiver Program, forYOU Team, Swaddle, and RISE. Psychological First Aid has been defined [42,43] as “a compassionate and supportive presence designed to reduce acute distress and facilitate access to continued care, if indicated” [42], p.1017. 

Several programs were also partly or fully based on Scott’s Three-Tiered Interventional Model of Second Victim Support [35] and/or adapted from the forYOU Team (YOU Matter Program, Second Victim Support Program at the Bali International Medical Centre Hospitals, Care for the Caregiver Program, RISE, Swaddle, Surgery-specific Second Victim Support Program). The latter [26] was in part also adapted from the Peer Support Service at Brigham and Women’s Hospital in Boston. 

Other models and toolkits serving as a conceptual foundation included the Just Culture Model [34] (Healing Beyond Today), the social resilience model [44] and G.R.A.C.E process (“Gathering attention, recalling intention, attuning, considering, engaging”) [45] (RISE), the Theory of Transpersonal Caring [46] (forYOU), the Communication and Optimal Resolution Toolkit [47] (Care for the Caregiver Program) and the Kaiser Permanente Model [48] (Peer Support Service). 

Moreover, literature reviews, expert panels, and pre-implementation surveys preceded the development of some of the support resources (Peer Support Team, forYOU Team, Swaddle, YOU Matter Program, MITSS Toolkits, MISE, Surgery-specific second victim support program, RISE). 

##### Incorporated Elements of Safety I and Safety II

While none of the included studies directly addressed the Safety I and/or Safety II approach [15], we identified certain aspects of the support resources corresponding to these approaches. 

The common goal of all support resources is to identify and to reduce second victims’ psychological distress due to the clinical event, thus reflecting a Safety I principle. At the same time, healthcare providers are seen as a resource for the healthcare system rather than as a source of errors, a view which is in accordance with the proactive Safety II approach. For instance, Morales and Brown [32] underline that “the emphasis is not on who made an error, but rather gaining insight into how clinicians made decisions in that instance” [32], p. 466. Indeed, resilient decision making may help clinicians to proactively identify issues and prevent adverse event occurrence. 

**Figure 4:**
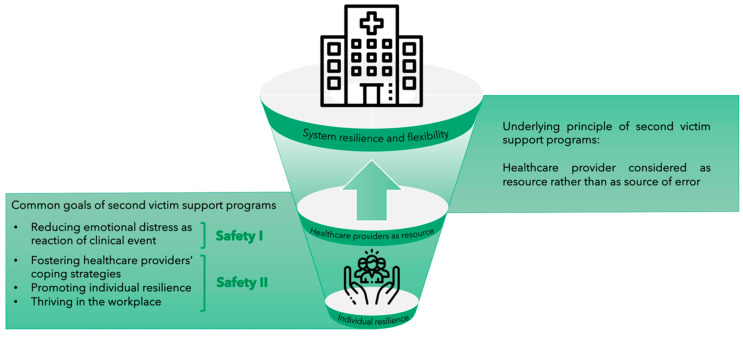
From individual resilience to system resilience and flexibility (Note: Icons made by Becris and Freepik from www.flaticon.com, accessed on 11 March 2021).

Moreover, some authors explicitly stated that their programs seek to foster and improve second victims’ coping strategies [26,29,32]. In the same vein, strengthening healthcare providers’ personal resilience is considered an important goal of the programs RISE, WUSM Peer Support Program, and Swaddle [23,24,25,29,36]. A concept similar to the one of individual resilience has been introduced by the founders of the forYOU Team, Scott and colleagues [49,50]. Indeed, “Thriving” is considered the most beneficial trajectory for second victims when “Moving on” after the stressful event [50]. By drawing on the Social Resilience Model, Connors et al. [23] highlighted that fostering individual resilience can even lead to institutional resilience. This vision is in line with the approach of systemic resilience and flexibility by Hollnagel et al. [15] (see Figure 4).

#### 3.2.3. Descriptive Statistics of Support Encounters

Five studies reported the number of support encounters since program inception [25,26,27,29,30]. The forYOU Team recorded 479 peer support encounters in the first 5 years, including 1028 healthcare providers [27]; RISE recorded 119 encounters in the first 52 months, including approximately 500 healthcare providers [25]; YouMatter recorded 253 encounters [30]; the Surgery-specific Second Victim Support Program at Massachusetts General Hospital recorded 47 outreach interventions in the first year; and the WUSM Peer Support Program recorded 165 individuals requesting support [29]. While two studies found a higher percentage of one-on-one encounters (82.7% [27], 91.7% [30]) than group encounters, Edrees et al. [25] recorded more group than individual sessions (56%). 

Information on average duration of the encounter was provided by two studies. The RISE and forYOU Team encounters lasted on average 24 min to 1 hour, respectively [25,27].

Requests for support were not only linked to adverse events (e.g., 21.3% of RISE encounters) and medical errors (e.g., 5% of RISE encounters, 2% of forYOU group encounters, 17% of forYOU one-on-one encounters) but also to other situations, such as patient death (e.g., 45% of RISE encounters), unanticipated patient outcome (e.g., 55% of forYOU one-on-one encounters, 65% of forYOU group encounters), personal/professional crisis (e.g., 33% of forYOU group encounters, 28% of forYOU one-on-one encounters), emotional distress and burnout, staff assault, difficult decisions, and intraoperative mishaps [25,26,27,30]. 

Data on additional support, such as referrals to other structures and follow-up, were mentioned by four studies. Referrals to higher levels of support (e.g., employee assistance program, clinical psychologist) were required in 6.4% [26] to 9.7% of the encounters [27,29]. Edrees et al. [25] documented that additional support sources were offered by 84.3% of RISE peer supporters. Hirschinger et al. [27] mentioned that follow-up was needed by one-third of the staff supported by forYOU. The WUSM Peer Support Program saw a median of 2 interactions, with the number of encounters ranging from 1 to 10 [29]. 

#### 3.2.4. Descriptive Statistics of Supported Staff and Peer Supporters

Four studies reported data on the professions of the supported staff [25,27,29,30]. The programs forYOU, RISE, YouMatter, and WUSM Peer Support Program supported a wide range of professions, such as nurses, physicians, residents, fellows, respiratory technicians, pharmacists, patient-care assistants, security staff, social workers, medics, paramedics, unit clerks, and nurses’ aides. In three of the four studies, nurses constituted the biggest group, with percentages ranging from 32.32% [30] to 56.3% [25]. 

Three studies provided descriptive statistics on peer supporters’ discipline [25,29,30], with nurses making up the largest group in the YouMatter Program (44%) [30] and in RISE (63.3%) [25]. 

### 3.3. Benefits of Support Programs for Second Victims

There is little empirical data on the beneficial effects and effectiveness of the established second victim support programs [24,25,26,30,31,33]. 

Results obtained from a postimplementation survey of the YouMatter program at the Nationwide Children’s Hospital showed that 85% of respondents considered the program beneficial for the department, with three healthcare workers stating that they personally benefited from the encounter [30]. Regarding RISE, two-thirds of peer supporters rated the success of peer encounters as excellent (66.7%), and more than 80% were confident to have met callers’ expectations (87.8%) and satisfied with the encounter (82.4%) [25]. Over 90% of participants (i.e., 93%) who had either used RISE or knew a colleague who had used it said that they would very likely recommend RISE to other colleagues [24]. Moreover, four years after the implementation of RISE, perception of availability and benefits of support was significantly greater than at baseline (*p* < 0.001 and *p* = 0.014, respectively). Qualitative analysis also revealed that callers considered the program useful [24]. Similarly, qualitative findings by El Hechi et al. [26] showed that both supporters and callers rated the Surgery-specific Second Victim Program at Massachusetts General Hospital positively. Further, both the MITTS Toolkit and the MISE website were positively evaluated by the majority of participants [31,33]. As regards the website, Mira et al. [31] also reported an increase of knowledge on patient safety issues and the second victim phenomenon among participants. 

There were two studies assessing the impact of the second victim support program on the workplace culture [26,38]. Namely, 81% of those surveyed said that the surgery-specific second victim support program had a positive impact on the department’s safety and support culture [26], and Wijaya et al. [38] demonstrated a significant increase in patient safety culture after the implementation of the second victim program at the Bali International Medical Centre Hospitals Kuta and Nusa. 

### 3.4. Personal Perceptions and Experiences of Peer Supporters

Only two studies [23,25], both focusing on the RISE program, assessed the personal experiences and perceptions of the involved peer supporters. Edrees et al. [25] reported that more than two-thirds of peer responders (approx. 70%) did not feel, or felt only slightly, emotionally distressed after an encounter with a second victim but some of the interviewed participants felt less confident and more distressed in group encounters because of less training for this type of support. Peer responders also worried about not being able to follow up with the callers after an encounter. 

A recent study by Connors et al. [23] found that more than 90% of RISE members considered their role as peer responders meaningful, satisfying, and positively impactful and more than 80% felt confident and autonomous in performing this role. While the majority felt emotionally resilient (56%), more than a quarter (28%) reported feelings of burnout from their tasks as RISE supporters. Further, respondents stated that they experienced greater energy and enjoyment, and felt empowered and a personal affinity with RISE. 

### 3.5. Challenges Encountered during Implementation of the Programs 

Several authors discussed challenges during the implementation of the programs [24,25,29,30,35,37]. 

**Figure 5:**
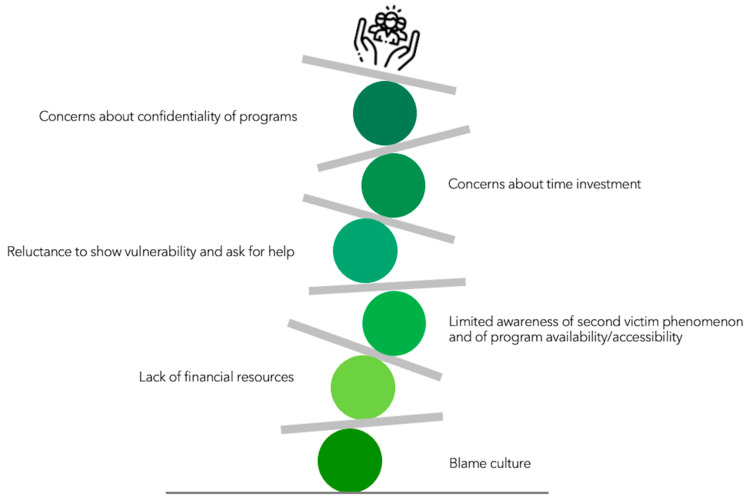
Challenges during program implementation (Note: Icon made by Freepik from www.flaticon.com, accessed on 11 March 2021).

As depicted in Figure 5, one of the encountered obstacles was limited awareness of the importance of the second victim phenomenon and of the availability and accessibility of support programs [24,25,30]. Another challenge in reaching out to distressed healthcare providers was a still-existing culture of blame and a reluctance in the healthcare community to show vulnerability and ask for help [24,37].

Concerns about the confidentiality of the program and potential legal risks both for supported staff and peer supporters represented another barrier when reaching out to affected staff and recruiting supporters [25,29]. Further, time investment was seen as problematic for (potential) peer supporters [24,29]. Additional challenges were a lack of financial resources as well as a lack of apparent financial rewards for the healthcare institutions [25,29,30]. 

## 4. Discussion

The findings from this systematic review shed new light on institution-based, formalized second victim support resources. Our search identified 12 support resources described in the academic literature and implemented in the past 15 years. One important finding was that most of these resources were established in the United States and that organized support interventions are still missing in most hospitals and medical centers around the world. 

Our formal narrative synthesis revealed several challenges research groups had to face during the implementation of the support programs. Some of these challenges were organizational in nature, such as concerns about time investment and limited awareness of program availability and accessibility. Other challenges, such as limited awareness of the second victim phenomenon, concerns about confidentiality of programs, reluctance by staff to show vulnerability and ask for help, as well as lack of funding are closely linked to the persisting culture in medicine that blames the individual healthcare provider when things go wrong, stigmatizes apparent weakness in healthcare staff, and does not see the imminent need to foster healthcare providers’ well-being [12,15,51,52,53,54]. 

As suggested by two of the included studies [26,38], second victim support programs may have a positive impact on the safety culture of healthcare institutions. Indeed, the implementation of a second victim support intervention is by itself a powerful statement against blame culture and stigmatization of mental issues and sends a signal to the entire healthcare workforce that their professional and personal well-being is important and that they are deserving of psychological support. Moreover, as indicated by our findings, common goals of the support programs, such as fostering healthcare providers’ coping strategies and promoting individual resilience, are consistent with the Safety II approach, which sees the healthcare provider as a resource for system resilience and flexibility [15,55] rather than as a source of error. As outlined earlier, the goal of support programs to reduce second victims’ distress as a reaction to the clinical event can be considered a Safety I principle. Indeed, it is mainly focused on identifying and trying to fix “what has gone wrong” in the healthcare provider as a consequence of having been involved in an adverse event. One could argue that by incorporating elements of both Safety I and Safety II, the support programs follow a path that was suggested by Hollnagel and colleagues: “The way forward therefore lies in combining the two ways of thinking” [15], p. 5.

While there has been little quantitative analysis of the beneficial effects of support programs on the affected staff, the extracted data point to the usefulness and success of the programs. In line with this, several programs applied psychological first aid, which has been described as “acute intervention of choice” [56], p. 5, in the aftermath of traumatic, stressful events, and is preliminarily shown to be effective [39,56,57]. 

Another interesting finding to emerge from this study was that support programs may have not only a positive impact on the affected staff but also on the peer responders. Indeed, one of the primary studies [23] found that peer responders see their role as a source of meaning, joy, and satisfaction. At the same time, peer supporters can sometimes experience distress and burnout related to their tasks [23,25]. As recently pointed out by Connors et al. [23], peer supporters’ well-being should be therefore monitored and feelings of distress or burnout arising from their role properly addressed and managed. 

### 4.1. Recommendations for Clinical Practice 

As the results of our systematic review underline, there are only a few second victim support interventions worldwide. Investing in such support structures should become a top priority for healthcare institutions striving for a just and transparent culture and system resilience [15,58]. 

Aside from support programs providing psychological first aid and immediate emotional support, we also suggest the need for programs offering medium- and long-term support for second victims. Such programs could better monitor healthcare providers’ professional and personal well-being and focus on the development and application of a set of coping strategies as adaptive response to the traumatic clinical event [5,11]. 

We also encourage hospitals with existing support programs to widely promote and inform about their interventions on websites, social media, and other communication channels to motivate other institutions to follow the lead. Increased visibility would also help to raise awareness of the resources available to healthcare providers, to overcome their concerns about confidentiality, to further destigmatize medical errors and the need for psychological help [12,53,54], and to recruit new peer supporters. Additionally, offering patient safety training for health profession students [59] and healthcare workers could help to deepen the understanding of the second victim phenomenon and other problematic clinical events. 

Existing support programs for second victims might also be successfully expanded to other types of clinical events. For instance, the programs RISE and forYOU were successfully extended to workplace violence support after an increase in referrals for exposure to violent events had been identified [39]. Further, in light of the current pandemic, established second victim support programs should be made available also to healthcare workers experiencing COVID-19-related distress and trauma, a step that could save organizational and financial resources [60,61]. 

Finally, as others have highlighted [37,62,63], to provide a “comprehensive emotional support response” [37], p. 252, for all involved stakeholders, healthcare institutions must also ensure timely and easily accessible psychological support for patients harmed by adverse events and their caregivers. 

### 4.2. Limitations

This study has several limitations. First, despite an extensive and highly structured search of the published and gray literature, we may have missed relevant papers. Second, since we restricted our search to support resources presented in academic articles, we did not describe all actually existing programs (e.g., Denver Health’s RISE program [64]). 

Third, some steps of the applied methodology, particularly data extraction, quality assessment, and formal narrative synthesis, can be prone to subjective judgment and lack of transparency. To address this weakness, each of these methodological steps were conducted by two independent reviewers/appraisers and carefully documented. Fourth, we were not able to provide a synthesis of the programs’ effectiveness due to a lack of sufficient data. Indeed, only a few studies provided preliminary data on the beneficial effects of the support programs. A possible explanation for this observation is that many of the primary papers only aimed to describe the development and implementation of the respective program. This may be also partly explained by the fact that the unique and highly confidential nature of peer support encounters makes a precise measurement of their effectiveness, including also follow-up data, very challenging, as pointed out in the literature [25,39]. Fifth, the included studies were heterogeneous in terms of article type, study design, applied questionnaires, time passed since program inception, and data description and analysis, thus impeding synthesis and detailed subgroup analyses. Sixth, many primary studies had small sample sizes and were performed at a single department or healthcare institution, thus limiting generalizability. Moreover, several included studies reported cross-sectional data collected from self-report questionnaires, being therefore susceptible to certain biases, such as recall bias and nonresponse bias. Finally, we could only provide a snapshot of the support programs as certain aspects (e.g., characteristics of supported staff and peer supporters) may have already changed since the publication of the included studies. 

### 4.3. Future Research Directions

Further research, determining the effectiveness of the support programs and tools without violating the confidentiality of the encounters, should be undertaken. Establishing an evidence base for the beneficial effects of these interventions on second victims’ mental health and applied coping strategies would build a strong argument that medical governing bodies and healthcare institutions should provide funds to implement second victim support structures on a large scale. Performing cost-benefit analyses as done by Moran et al. [65] could help in this endeavor. 

It would be also interesting to better understand if the type (group vs. one-on-one encounters) and timing and duration of support (e.g., immediately after the event, several weeks later, continuous monitoring over several weeks) affects different outcomes, such as emotional distress, absenteeism, and coping skills. Future studies should also assess if the availability of a second victim support structure positively influences the error disclosure practices and patient safety attitudes of the healthcare staff, the delivered quality of care, and the overall safety culture of the healthcare institution [24,33,38]. 

Additional investigations are needed to explore peer supporters’ challenges and motivations as well as the positive and negative impact this role might have on their overall professional performance, their psychological well-being, and resilience. As noted by Conners et al. [23], such studies would be instrumental in drawing attention to and promoting the role of peer supporters as an important part of a positive safety culture and a resilient healthcare system. 

Finally, second victim support programs that have been expanded to other types of clinical events or to other medical settings should be carefully described in future studies. Given the COVID-19 crisis, it should also be evaluated how successful support structures originally designed for second victims can be used in mitigating the strong psychological impact of this pandemic on healthcare providers’ mental health [61,66]. 

## 5. Conclusions

This systematic review provides a detailed overview of 12 institution-based, formalized second victim support resources described in the scientific literature, highlighting that, except for the United States, second victim support structures are still rare in most countries. This study also advances our understanding of implementation challenges, such as a still-existing blame culture, lack of financial resources, and reluctance among healthcare providers to show vulnerability and ask for help, as well as a limited awareness of the second victim phenomenon and of program availability and accessibility. Beneficial effects of the programs were identified for workplace safety and support culture in general, and for the affected staff as well as the peer responders in particular. The latter group considered their role highly gratifying and joyful but reported in some cases also symptoms of distress and burnout related to their role as peer supporters. Our findings point to the need for a strong investment in the implementation of second victim support structures offering immediate, medium-, and long-term support, for an increased promotion of already existing support resources, as well as for a monitoring of peer supporters’ well-being. 

Common goals of the support programs, such as reducing emotional distress as a reaction to the stressful clinical event, fostering healthcare providers’ coping strategies, and promoting individual resilience, may then act as a basis for long-term systemic resilience and flexibility. 

## Figures and Tables

**Table 1:** Risk of bias assessment of included studies.

MMAT TOOL											
**Qualitative Studies**											
		1. Is the qualitative approach appropriate to answer the research question?		2. Are the qualitative data collection methods adequate to address the research question?		3. Are the findings adequately derived from the data?		4. Is the interpretation of results sufficiently substantiated by data?		5. Is there coherence between qualitative data sources, collection analysis, and interpretation?	
Trent et al., 2016 [36] (SWADDLE)		✓		✓		✓		✓		✓	
**Quantitative non-randomized studies**											
		1. Are the participants representative of the target population?		2. Are measurements appropriate regarding both the outcome and intervention (or exposure)?		3. Are there complete outcome data?		4. Are the confounders accounted for in the design and analysis?		5. During the study period, is the intervention administered (or exposure occurred) as intended?	
Wijaya et al., 2018 [38] (no specific name given)		✓		✓		✗		✓		✓	
**Quantitative descriptive studies**											
		1. Is the sampling strategy relevant to address the research question?		2. Is the sample representative of the target population?		3. Are the measurements appropriate?		4. Is the risk of nonresponse bias low? (for case series and case report: are there complete data on the cases?)		5. Is the statistical analysis appropriate to answer the research question?	
Hirschinger et al., 2015 [27] (forYOU Team)		✓		✓		✓		✓		✓	
Krzan et al., 2015 [28] (YouMatter Program)		✓		✓		✓		✓		✗	
Lane et al., 2018 [29] (WUSM Peer Support Program)		✓		✓		✓		✗		✗	
Merandi et al., 2017 [30] (YouMatter Program)		✓		✓		✓		✓		✗	
Mira et al., 2017 [31] (MISE)		✓		✓		✓		✓		✗	
**Mixed methods studies**											
		1. Is there an adequate rationale for using a mixed methods design to address the research question?		2. Are the different components of the study effectively integrated to answer the research questions?		3. Are the outputs of the integration of qualitative and quantitative components adequately interpreted?		4. Are divergences and inconsistencies between quantitative and qualitative results adequately addressed?		5. Do the different components of the study adhere to the quality criteria of each tradition of the methods involved?	
Connors et al., 2021 [23] (RISE)		✓		✓		✓		✓		✓	
Dukhanin et al., 2018 [24] (RISE)		✓		✓		✓		✓		✓	
Edrees et al., 2016 [25] (RISE)		✓		✓		✓		✓		✓	
El Hechi et al., 2019 [26] (Surgery-Specific Second Victim Support Program)		✓		✓		✓		✓		✗	
Scott et al., 2010 [35] (forYOU Team)		✓		✓		✓		✓		✓	
**JBI CRITICAL APPRAISAL CHECKLIST FOR TEXT AND OPINION PAPERS**											
	1. Source of opinion identified		2. Source of opinion having a standing in the field		3. Interests of the relevant population as central focus of the opinion		4. Stated position as result of analytical process and logic in the expressed opinion		5. Reference to the extant literature		6. Incongruence with the literature/sources logically defended
Morales & Brown, 2019 [32] (Care for the Caregiver Program)	✓		✓		✓		✓		✓		✓
Pratt et al., 2012 [33] (Medically Induced Trauma Support Services Tool)	✓		✓		✓		✓		✓		✓
Roesler et al., 2009 [34] (Healing Beyond Today)	✓		✓		✓		✓		✗		✗
Van Pelt, 2008 [37] (Peer Support Team)	✓		✓		✓		✓		✓		✓

✓= Yes; ✗= No; ? = Unclear.

## Data Availability

The data presented in this study are available on reasonable request from the corresponding author.

## Supplementary Materials

The following are available online at https://www.mdpi.com/article/10.3390/ijerph18105080/s1. Supplementary File S1: Search strategy and retrieved records from each electronic database; Supplementary File S2: Additional Searches; Supplementary File S3: List of excluded studies; Supplementary File S4: Summary of second victim support programs as described in the primary studies.
