# Supplementary material for: Promoting the Psychological Well-Being of Healthcare Providers Facing the Burden of Adverse Events: A Systematic Review of Second Victim Support Resources
Source: Int J Environ Res Public Health. 2021 May 11;18(10):5080. doi: 10.3390/ijerph18105080 (PMC8151650; doi:10.3390/ijerph18105080)
Supplement: Supplementary file 1 [file ijerph-18-05080-s001.zip › Supplementary File 1.pdf]

SUPPLEMENTARY FILE 1. SEARCH STRATEGY AND RETRIEVED RECORDS FROM EACH ELECTRONIC DATABASE

1. **PubMed**
   - Date of search: 13/12/2019
   - Search strategy:
   - (medical error OR patient safety incident OR adverse event OR near miss OR human error) AND (health personnel OR second victim OR health professional OR health care provider OR nurse OR physician OR clinician) AND (intervention OR support program OR support strategy OR support protocol OR support system)
   - *n* findings: 1140

2. **MEDLINE**
   - Date of search: 13/12/2019
   - Search strategy: (medical error OR patient safety incident OR adverse event OR near miss OR human error) AND (health personnel OR second victim OR health professional OR health care provider OR nurse OR physician OR clinician) AND (intervention OR support program OR support strategy OR support protocol OR support system)
   - *n* findings: 1667

3. **Scopus**
   - Date of search: 13/12/2019
   - Search strategy: (medical error OR patient safety incident OR adverse event OR near miss OR human error) AND (health personnel OR second victim OR health professional OR health care provider OR nurse OR physician OR clinician) AND (intervention OR support program OR support strategy OR support protocol OR support system)
   - *n* findings: 36

4. **Web of science**
   - Date of search: 18/12/2019
   - Search strategy: (medical error OR patient safety incident OR adverse event OR near miss OR human error) AND (health personnel OR second victim OR health professional OR health care provider OR nurse OR physician OR clinician) AND (intervention OR support program OR support strategy OR support protocol OR support system)
   - *n* findings: 489

5. **ScienceDirect**
   - Date of search: 13/12/2019
   - Search strategy: (medical error OR patient safety incident OR adverse event OR near miss OR human error) AND (health personnel OR second victim OR health professional OR health care provider OR nurse OR physician OR clinician) AND (intervention OR support program OR support strategy OR support protocol OR support system)
   - *n* findings: 131

6. **PsycINFO**
   - Date of search: 13/12/2019
   - Search strategy: (medical error OR patient safety incident OR adverse event OR near miss OR human error) AND (health personnel OR second victim OR health professional OR health care provider OR nurse OR physician OR clinician) AND (intervention OR support program OR support strategy OR support protocol OR support system)
   - *n* findings: 12
