# Supplementary material for: Promoting the Psychological Well-Being of Healthcare Providers Facing the Burden of Adverse Events: A Systematic Review of Second Victim Support Resources
Source: Int J Environ Res Public Health. 2021 May 11;18(10):5080. doi: 10.3390/ijerph18105080 (PMC8151650; doi:10.3390/ijerph18105080)
Supplement: Supplementary file 1 [file ijerph-18-05080-s001.zip › Supplementary File 2.pdf]

SUPPLEMENTARY FILE 2. ADDITIONAL SEARCHES

Email search alerts

Automatic weekly email search alerts were set up for the database Web of Science and PubMed from December 18, 2019 – January 20, 2021.

Grey databases

1. **OpenSIGLE database/opengrey.eu**
   - Date of the search: 10/07/2020
   - Search strategy: (medical error OR patient safety incident OR adverse event OR near miss OR human error) AND (health personnel OR second victim OR health professional OR health care provider OR nurse OR physician OR clinician) AND (intervention OR support program OR support strategy OR support protocol OR support system)
   - Retrieved records: n= 2

2. **PsycEXTRA**
   - Date of the search: 10/07/2020
   - Search strategy: (medical error OR patient safety incident OR adverse event OR near miss OR human error) AND (health personnel OR second victim OR health professional OR health care provider OR nurse OR physician OR clinician) AND (intervention OR support program OR support strategy OR support protocol OR support system)
   - Retrieved records: n= 41

3. **Grey Literature Project (http://www.greylit.org/home)**

   - Date of the search: 10/07/2020
   - Since the complete search strategy did not reveal any findings, a search was conducted with the following search term: "second victim program"
   - Retrieved records: n=4

Reference lists of white papers, consensus statements, books

- Conway J, Federico F, Stewart K, Campbell MJ. Respectful Management of Serious Clinical Adverse Events (Second Edition). IHI Innovation Series white paper. Cambridge, Massachusetts: Institute for Healthcare Improvement; 2011. (Available on www.IHI.org)
- Dekker S. Second Victim. Error, Guilt, Trauma and Resilience. Boca Raton, FL: CRC Press, Taylor & Francis Group, 2013.

- Perlo J, Balik B, Swensen S, Kabcenell A, Landsman J, Feeley D. IHI Framework for Improving Joy in Work. IHI White Paper. Cambridge, Massachusetts: Institute for Healthcare Improvement; 2017. (Available at ihi.org)
- National Patient Safety Foundation's Lucian Leape Institute. Through the eyes of the workforce. Creating joy, meaning, and safer health care. Boston, MA: National Patient Safety Foundation; 2013.
- When Things Go Wrong: Responding to Adverse Events. A Consensus Statement of the Harvard Hospitals. Burlington, MA: Massachusetts Coalition for the Prevention of Medical Errors; March 2006. Available at: http://www.macoalition.org/documents/respondingToAdverseEvents.pdf.

Reference lists of reviews

- Brasaite I, Kaunonen M, Suominen T. Healthcare professionals' knowledge, attitudes and skills regarding patient safety: a systematic literature review. Scand J Caring Sci 2015;29:30- 50.
- Chan ST, Khong PCB, Wang W. Psychological responses, coping and supporting needs of healthcare professionals as second victims. Int Nurs Rev 2017;64:242-62.
- Coughlan B, Powell D, Higgins MF. The second victim: a review. Eur J Obstet Gynecol Reprod Biol 2017;213:11-16.
- Lewis EJ, Baernholdt M, Hamric AB. Nurses' experience of medical errors: an integrative literature review. J Nurs Care Qual 2013;28:153–61.
- Nydoo P, Pillay BJ, Naicker T, Moodley J. The second victim phenomenon in health care: A literature review. Scand J Public Health. 2020;48(6):629-637. doi:10.1177/1403494819855506
- Schwappach DL, Boluarte TA. The emotional impact of medical error involvement on physicians: a call for leadership and organizational accountability. Swiss Med Wkly 2009;139:9-15.
- Seys D, Wu AW, Van Gerven E, et al. Health care professionals as second victims after adverse events: a systematic review. Eval Health Prof 2013;36:135-62.
- Seys D, Scott S, Wu A, et al. Supporting involved health care professionals (second victims) following an adverse health event: a literature review. Int J Nurs Stud 2013;50:678-87.
- Sirriyeh R, Lawton R, Gardner P, et al. Coping with medical error: a systematic review of papers to assess the effects of involvement in medical errors on healthcare professionals' psychological well-being. Qual Saf Health Care 2010;19:e43.
- Stone, Misty PhD, RN Second victim support programs for healthcare organizations, Nursing Management (Springhouse): June 2020 - Volume 51 - Issue 6 - p 38-45 doi: 10.1097/01.NUMA.0000662664.90688.1d
- Wade, Laura MD*; Fitzpatrick, Eleanor MN, RN†; Williams, Natalie BScN*; Parker, Robin MLIS‡; Hurley, Katrina F. MD, MHI, FRCPC† Organizational Interventions to Support Second Victims in Acute Care Settings, Journal of Patient Safety: May 13, 2020 - Volume Publish Ahead of Print – Issue. doi: 10.1097/PTS.0000000000000704
- White AA, Waterman AD, McCotter P, et al. Supporting health care workers after medical error: considerations for health care leaders. JCOM 2008;15:240-47.
- Wu AW, Shapiro J, Harrison R, et al. The impact of adverse events on clinicians: what's in a name? J Patient Saf Published Online First: November 4, 2017.doi:10.1097/PTS.0000000000000256

Volumes of Journals (January 2010 – August 2020)

- Journal of Patient Safety
- Joint Commission Journal on Quality and Patient Safety
- BMJ Quality and Safety (formerly Quality in Health Care and Quality & Safety in Health Care)

Search of websites dedicated to second victims

- https://betsylehmancenterma.gov/initiatives/clinician-support?utm_source=mitss&utm_medium=website&utm_campaign=redirect
- https://www.centerforpatientsafety.org/second-victims/
- http://www.secondvictim.be/index.html
- https://www.rn.com/headlines-in-health/supporting-second-victims/
- https://www.rcem.ac.uk/docs/Safety/82e.%20Supporting-the-Second-Victim.pdf
