# Supplementary material for: Promoting the Psychological Well-Being of Healthcare Providers Facing the Burden of Adverse Events: A Systematic Review of Second Victim Support Resources
Source: Int J Environ Res Public Health. 2021 May 11;18(10):5080. doi: 10.3390/ijerph18105080 (PMC8151650; doi:10.3390/ijerph18105080)
Supplement: Supplementary file 1 [file ijerph-18-05080-s001.zip › Supplementary File 3.pdf]

SUPPLEMENTARY FILE 3. LIST OF EXCLUDED STUDIES

Excluded studies

Study		Reason for exclusion
1	Arnold, J.; Tango, J.; Walker, I.; Waranch, C.; McKamie, J.; Poonja, Z.; Messman, A. An Evidence-based, Longitudinal Curriculum for Resident Physician Wellness: The 2017 Resident Wellness Consensus Summit. *West J Emerg Med.* **2018** Mar; *19*:337–341.	Mismatch with inclusion criteria
2	Baker, R.G. Governance, policy and system-level efforts to support safer healthcare. *Healthc. Q.* **2014**, *17*, 21–6.	Wrong focus
3	Baylor Scott and White Medical Center—Temple. House Staff Handbook [PDF]. January 2018. Available online: https://www.bswhealth.med/education/Documents/temple/house-staff-handbook.pdf	Mismatch with inclusion criteria
4	Bell, S.K.; Moorman, D.W.; Delbanco, T. Improving the patient, family, and clinician experience after harmful events: the "when things go wrong" curriculum. *Acad. Med.* **2010**, *85*, 1010–1017.	Mismatch with inclusion criteria
5	Berman, L.; Rialon, KL.; Mueller, CM.; Ottosen, M.; Weintraub, A.; Coakley, B.; Brandt, ML; Heiss, K. Supporting recovery after adverse events: An essential component of surgeon well-being. *J. Pediatr. Surg.* **2021**, doi: 10.1016/j.jpedsurg.2020.12.031.	Mismatch with inclusion criteria
6	Biggs, S.; Waggett, HB.; Shabbir, J. Impact of surgical complications on the operating surgeon. *Colorectal. Dis.* **2020**, *22*, 1169–1174.	Mismatch with inclusion criteria
7	Brunelli, M.V.; Estrada, S.; Celano, C. Cross-Cultural Adaptation and Psychometric Evaluation of a Second Victim Experience and Support Tool (SVEST). *J. Patient. Saf.* **2018**, doi: 10.1097/PTS.0000000000000497.	Wrong focus
8	Bueno Domínguez, M.; Briansó Florencio, M.; Colomés Figuera, L., et al. Experiencia de unidad de soporte a las segundas víctimas (USVIC). *Rev. Calid. Asis.* **2016**, *31*, 49–52.	Mismatch with inclusion criteria
9	Butt, T.F.; Cox, A.R.; Lewis, H.; Ferner, R.E. Patient experiences of serious adverse drug reactions and their attitudes to medicines: a qualitative study of survivors of Stevens-Johnson syndrome and toxic epidermal necrolysis in the UK. *Drug. Safety.* **2011**, *34*, 319–328.	Mismatch with inclusion criteria
10	Chen, C.C.; Chen, K.; Hsu, C.Y.; Li, Y.C. Developing guideline-based decision support systems using protégé and jess. *Comput. Methods. Programs. Biomed.* **2011**, *102*, 288–94.	Mismatch with inclusion criteria
11	Cheng, S.T.; Tsui, P.K.; Lam, J.H. Improving mental health in health care practitioners: randomized controlled trial of a gratitude intervention. *J. Consult. Clin. Psychol.* **2015**, *83*, 177–186.	Wrong focus
12	Christoffersen, L.; Teigen, J.; Rønningstad, C. Following-up midwives after adverse incidents: How front-line management practices help second victims. *Midwifery.* **2020**, *85*,102669.	Wrong focus
13	Clancy, C.M. Alleviating "second victim" syndrome: how we should handle patient harm. *J. Nurs. Care. Qual.* **2012** *27*, 1–5.	Mismatch with the inclusion criteria

14	Choi, N.; McGoldrick, J.; Borgetti, S. Creating debriefing sessions for perceived medical errors in residency: a step towards wellness. *J. Gen. Intern. Med*. **2017**, *32*, 83–808.	Full-text not available
15	Christoffersen, L.; Teigen, J.; Rønningstad, C. Following-up midwives after adverse incidents: How front-line management practices help second victims. *Midwifery*. **2020**, *85*,102669.	Wrong focus
16	Chung AS, Smart J, Zdradzinski M, Roth S, Gende A, Conroy K, Battaglioli N. Educator Toolkits on Second Victim Syndrome, Mindfulness and Meditation, and Positive Psychology: The 2017 Resident Wellness Consensus Summit. *West. J. Emerg. Med*. **2018,** *19*, 327–331.	Mismatch with inclusion criteria
17	Daniels, R.G.; McCorkle, R. Design of an evidence-based "second victim" curriculum for nurse anesthetists. *AANA. J.* **2016**, *84*, 107–113.	Full-text not available
18	Devencenzi, T., O'Keefe, J. To err is human: supporting the patient care provider in the aftermath of an unanticipated adverse clinical outcome. *Int. J. Emerg. Ment. Health.* **2006***, 8,* 131–135.	Full-text not available
19	de Wit, M.E.; Marks, C.M.; Natterman, J.P.; Wu AW. Supporting second victims of patient safety events: shouldn't these communications be covered by legal privilege? *J. Law. Med. Ethics.* **2013***, 41,* 852–8.	Wrong focus
20	Edrees H.; Brock, D.M.; Wu, A.W; McCotter, P.I.; Hofeldt, R.; Shannon, S.E.; Gallagher, T.H.; White, A.A. The experiences of risk managers in providing emotional support for health care workers after adverse events. *J. Healthc. Risk. Manag*. **2016**, *35,* 14–21.	Wrong focus
21	Edrees, H.H.; Morlock, L.; Wu, A.W. Do Hospitals Support Second Victims? Collective Insights From Patient Safety Leaders in Maryland. *Jt. Comm. J. Qual. Patient. Saf*. **2017**, *43*, 471–483.	Wrong focus
22	Edrees, H.H.; Wu, A.W. Does One Size Fit All? Assessing the Need for Organizational Second Victim Support Programs. *J. Patient. Saf*. **2017**, doi: 10.1097/PTS.0000000000000321.	Wrong focus
23	Egger, E. It pays for hospitals to support programs that help control physician stress. *Health. Care. Strateg. Manage*. **2000**, *18*,18–9.	Mismatch with inclusion criteria
24	Epstein, R.M.; Krasner, M.S. Physician resilience: what it means, why it matters, and how to promote it. *Acad. Med.* **2013**, *3*, 301–303.	Mismatch with inclusion criteria
25	Finney, R.E.; Torbenson, V.E.; Riggan, K.A.; Weaver, A.L.; Long, M.E.; Allyse, M.A.; Rivera-Chiauzzi, E.Y. Second victim experiences of nurses in obstetrics and gynaecology: A Second Victim Experience and Support Tool Survey. *J. Nurs. Manag.* **2020**, doi: 10.1111/jonm.13198.	Mismatch with inclusion criteria
26	Gamble, B.; Gamble, K.J. A systems approach to address the impact of second victim phenomenon. *Health. Serv. Manage. Res*. **2020**, doi: 10.1177/0951484820971455.	Mismatch with inclusion criteria
27	Gatzert, M.; Wobbe, A.; York, L. *We Care Team: Caring for the Care Giver*. Barnes-Jewish Hospital: St. Louis, MO, USA, 2015.	Full-text not available
28	Gomes, C.A. Practitioner application: RISE: Exploring Volunteer Retention and Sustainability of a Second Victim Support Program. *J. Healthc. Manag.* **2021***, 66,* 31–32.	Mismatch with inclusion criteria
29	Graham, P.; Zerbi, G.; Norcross, W.; Montross-Thomas, L.; Lobbestael, L.; Davidson, J. Testing of A Caregiver Support Team. *Explore. (NY).* **2019**, *15,* 19–26.	Mismatch with inclusion criteria

30	Hall, L.W.; Scott, S.D. The second victim of adverse health care events. *Nurs. Clin. North. Am*. **2012,** *47,* 383–93.	Wrong focus
31	Han, K.; Bohnen, J.D.; Peponis, T.; Martinez, M.; Nandan, A.; Yeh, D.D.; Lee, J.; Demoya, M.; Velmahos, G.; Kaafarani, H.M.A. The Surgeon as the Second Victim? Results of the Boston Intraoperative Adverse Events Surgeons' Attitude (BISA) Study. *J. Am. Coll. Surg*. **2017**, *224*, 1048–1056.	Wrong focus
32	Harrison, R.; Wu, A. Critical incident stress debriefing after adverse patient safety events. *Am. J. Manag. Care*. **2017**, *23*, 310–312.	Mismatch with inclusion criteria
33	Hauk L. Support strategies for health care professionals who are second victims. *AORN. J*. **2018**,*107,* P7-P9.	Wrong focus
34	Health PEI. Critical Incident Staff Support (CISS) Managers' Toolkit. Version December 2014. Available online: http://www.gov.pe.ca/photos/original/src_ciss_tkit.pdf	Mismatch with inclusion criteria
35	Huang, H.; Chen, J.; Xiao, M.; Cao, S.; Zhao, Q. Experiences and responses of nursing students as second victims of patient safety incidents in a clinical setting: a mixed methods study. *J. Nurs. Manag*. **2020**, doi: 10.1111/jonm.13085.	Mismatch with inclusion criteria
36	Heard, G.C.; Thomas, R.D.; Sanderson, P.M. In the Aftermath: Attitudes of Anesthesiologists to Supportive Strategies After an Unexpected Intraoperative Patient Death. *Anesth. Analg*. **2016**,*122,* 1614–1624.	Wrong focus
37	Hoysted, C.; Jobson, L.; Alisic, E. A pilot randomized controlled trial evaluating a web-based training program on pediatric medical traumatic stress and trauma-informed care for emergency department staff. *Psychol. Serv*. **2019**,*16*, 38–47.	Wrong focus
38	Joesten, L.; Cipparrone, N.; Okuno-Jones, S.; DuBose, E.R. Assessing the perceived level of institutional support for the second victim after a patient safety event. *J Patient Saf*. **2015***, 11,* 73–78.	Mismatch with inclusion criteria
39	Johnson, B. Code Lavender: initiating holistic rapid response at the Cleveland Clinic. *Beginnings*. **2014***, 34,* 10–11.	Full-text not available
40	Kable, A.K.; Spigelman, A.D. Why clinicians involved with adverse events need much better support. *IJHG*. **2018**, *23,* 312–315.	Wrong focus
41	Kerkman, T.; Dijksman, L.M.; Baas, M.A.M.; Evers, R.; van Pampus, M.G.; Stramrood, C.A.I. Traumatic Experiences and the Midwifery Profession: A Cross-Sectional Study Among Dutch Midwives. *J. Midwifery. Womens. Health*. **2019**, *64,* 435–442.	Wrong focus
42	Khan, A.; Vinson, A.E. Physician Well-Being in Practice. *Anesth. Analg*. **2020***, 131,* 1359–1369.	Mismatch with inclusion criteria
43	Kim, E.M.; Kim, S.A.; Lee, J.R.; Burlison, J.D.; Oh, E.G. Psychometric Properties of Korean Version of the Second Victim Experience and Support Tool (K-SVEST). *J. Patient. Saf*. **2020***, 16,* 179–186.	Mismatch with inclusion criteria
44	Kruper, A.; Domeyer-Klenske, A.; Treat, R.; Pilarski, A.; Kaljo, K. Secondary Traumatic Stress in Ob-Gyn: A Mixed Methods Analysis Assessing Physician Impact and Needs. J Surg Educ. **2020,** doi: 10.1016/j.jsurg.2020.08.038.	Mismatch with inclusion criteria
45	Lazarus, A. Traumatized by practice: PTSD in physicians. *J. Med. Pract. Manage*. **2014***, 30,* 131–134.	Mismatch with inclusion criteria

46	Langham, M.R. Commentary on supporting recovery after adverse events: An essential component of surgeon well-being. *J. Pediatr. Surg.* **2021**, doi: 10.1016/j.jpedsurg.2021.01.006.	Mismatch with inclusion criteria
47	Loren, D.J.; Garbutt, J.; Dunagan, W.C.; Bommarito, K.M.; Ebers, A.G., Levinson, W.; Waterman, A.D.; Fraser, V.J.; Summy, E.A.; Gallagher, T.H. Risk managers, physicians, and disclosure of harmful medical errors. *Jt. Comm. J. Qual. Patient. Saf.* **2010**, *36*,101–108.	Wrong focus
48	Lynch, G. *Operational Report of the Critical Incident Stress Management Program of First Nations and Inuit Health Branch*. Wingate Consulting: Kanata, Ontario, Canada, 2004.	Full-text not available
49	Margulies, S.L.; Benham, J.; Liebermann, J.; Amdur, R.; Gaba, N.; Keller, J. Adverse Events in Obstetrics: Impacts on Providers and Staff of Maternity Care. *Cureus*. **2020**, *12,* e6732.	Mismatch with inclusion criteria
50	Manser, T. Managing the aftermath of critical incidents: meeting the needs of health-care providers and patients. *Best. Pract. Res. Clin. Anaesthesiol.* **2011**, *25*, 169–179.	Wrong focus
51	Marmon, L.M.; Heiss, K. Improving surgeon wellness: The second victim syndrome and quality of care. *Semin. Pediatr. Surg*. **2015**, *24,* 315–318.	Wrong focus
52	McDaniel, L.R.; Morris, C. The Second Victim Phenomenon: How Are Midwives Affected?. *J. Midwifery. Womens. Health*. **2020**, *65,* 503–511.	Mismatch with inclusion criteria
53	McGinley, P. MITSS: Supporting Patients and Families for More Than a Decade [Internet]. 2013. Available online: https://www.psqh.com/analysis/mitss-supporting-patients-and-families-for-more-than-a-decade/ (accessed April 24, 2020)	Wrong focus
54	McNeely, E. The consequences of job stress for nurses' health: time for a check-up. *Nurs. Outlook*. **2005**, *53,* 291–299.	Wrong focus
55	Millenson, M.L. The silence. *Health. Affairs*. **2003**, *22,* 103–112.	Wrong focus
56	Mira, J.J.; Lorenzo, S.; Carrillo, I.; Ferrús, L.; Pérez-Pérez, P.; Iglesias, F.; Silvestre, C.; Olivera, G.; Zavala, E.; Nuño-Solinís, R.; et al.; Research Group on Second and Third Victims. Interventions in health organisations to reduce the impact of adverse events in second and third victims. *BMC. Health. Serv. Res*. **2015**,*15,* 341.	Wrong focus
57	Moffatt-Bruce, S.D.; Nguyen, M.C.; Steinberg, B.; Holliday, S.; Klatt, M. Interventions to Reduce Burnout and Improve Resilience: Impact on a Health System's Outcomes. *Clin. Obstet. Gynecol*. **2019**, *62,* 432–443.	Mismatch with inclusion criteria
58	Mok, W.Q.; Chin, G.F.; Yap, S.F.; Wang W. A cross-sectional survey on nurses' second victim experience and quality of support resources in Singapore. *J Nurs Manag*. **2020**, *28,* 286–293.	Wrong focus
59	Monteverde S. [Affrontare le "seconde vittime" come compito etico organizzativo]. 2017	Wrong focus
60	Nicol, N. Support for "second victims". *Am. J. Health. Syst. Pharm.* **2015**,*72,* 523.	Mismatch with inclusion criteria
61	Olson, K.; Marchalik, D.; Farley, H.; Dean, S.M.; Lawrence, E.C.; Hamidi, M.S.; Rowe, S.; McCool, J.M.; O'Donovan, C.A.; Micek, M.A.; Stewart, M.T. Organizational strategies to reduce physician burnout and improve	Wrong focus

	professional fulfillment. *Curr. Probl. Pediatr. Adolesc. Health. Care*. **2019**, *49,* 100664.	
62	Paparella, S. Caring for the caregiver: moving beyond the finger pointing after an adverse event. *J. Emerg. Nurs*. **2011***, 37,* 263–265.	Wrong focus
63	Plews-Ogan, M.; May, N.; Owens, J. Ardelt, M.; Shapiro, J.; Bell, S.K. Wisdom in Medicine: What Helps Physicians After a Medical Error? *Acad. Med.* **2016**, *91,* 233–241.	Wrong focus
64	Pratt, S.D.; Jachna, B.R. Care of the clinician after an adverse event. *Int. J. Obstet. Anesth*. **2015**, *24*, 54–63.	Wrong focus
65	Quillivan, R.R.; Burlison, J.D.; Browne, E.K.; Scott, S.D.; Hoffman, JM. Patient Safety Culture and the Second Victim Phenomenon: Connecting Culture to Staff Distress in Nurses. *Jt. Comm. J. Qual. Patient. Saf.* **2016***, 42,* 377–386.	Wrong focus
66	Reiser Crelier, F.; Schwappach, D.; Schwendimann, R. Supporting health professionals after an adverse event in Swiss hospitals: a cross-sectional study. Swiss. Med. Wkly. **2020***, 150,* w20278.	Wrong focus
67	Rivera-Chiauzzi, E.Y.; Smith, H.A.; Moore-Murray, T.; Lee, C.; Goffman, D.; Bernstein, P.S.; Chazotte, C. Healing Our Own: A Randomized Trial to Assess Benefits of Peer Support. *J. Patient. Saf.* **2020**, doi: 10.1097/PTS.0000000000000771.	Full-text not available
68	Rowe M. Doctors' responses to medical errors. *Crit. Rev. Oncol. Hematol.* **2004***, 52,* 147–163.	Mismatch with inclusion criteria
69	Russ, M.J. Correlates of the Third Victim Phenomenon. *Psychiatr. Q.* **2017***,* 88, 917–920.	Wrong focus
70	Santomauro, C.M.; Kalkman C.J.; Dekker, S.W.A. Second victims, organizational resilience and the role of hospital administration. Journal of Hospital Administration **2014***, 3,* 95–103.	Mismatch with inclusion criteria
71	Schiechtl, B.; Hunger, M.S.; Schwappach, D.L.; Schmidt, C.E.; Padosch, S.A. "Second victim": "Critical incident stress management" in der klinischen Medizin [Second victim : Critical incident stress management in clinical medicine]. *Anaesthesist*. **2013**, *62,* 734–741.	Wrong focus
72	Schrøder, K.; Janssens, A.; Hvidt, E.A. Adverse events as transitional markers - Using liminality to understand experiences of second victims. *Soc. Sci. Med.* **2021***, 268,* 113598.	Mismatch with inclusion criteria
73	Schwappach, D.L.B. [Nach dem Behandlungsfehler Umgang mit Patienten, Angehörigen und dem involvierten Personal]. 2014<br>Schwappach DL. Nach dem Behandlungsfehler : Umgang mit Patienten, Angehörigen und dem involvierten Personal [In the aftermath of medical error: caring for patients, family, and the healthcare workers involved]. *Bundesgesundheitsblatt Gesundheitsforschung Gesundheitsschutz*. **2015**, *58*, 80–86.	Wrong focus
74	Seys, D.; Scott, S.; Wu, A.; Van Gerven, E.; Vleugels, A.; Euwema, M.; Panella, M.; Conway, J.; Sermeus, W.; Vanhaecht, K. Supporting involved health care professionals (second victims) following an adverse health event: a literature review. *Int. J. Nurs. Stud.* **2013***, 50,* 678–87.	Wrong focus
75	Shanafelt, T.D.; Lightner, D.J.; Conley, C.R.; Petrou, S.P.; Richardson, J.W.; Schroeder, P.J.; Brown, W.A. An Organization Model to Assist Individual	Wrong focus

	Physicians, Scientists, and Senior Health Care Administrators With Personal and Professional Needs. *Mayo Clin Proc*. **2017***, 92,* 1688–1696.	
76	Shanafelt, T.D.; Bradley, K.A.; Wipf, J.E.; Back, A.L. Burnout and self-reported patient care in an internal medicine residency program*. Ann Intern Med.* **2002***, 136,* 358–67.	Wrong focus
77	Shapiro, J.; Whittemore, A.; Tsen, L.C. Instituting a culture of professionalism: the establishment of a center for professionalism and peer support. *Jt Comm J Qual Patient Saf.* **2014**, *40,* 168–177.	Wrong focus
78	Shapiro, J.; Galowitz, P. Peer Support for Clinicians: A Programmatic Approach. *Acad. Med.* **2016***, 91,* 1200–1204.	Mismatch with inclusion criteria
79	Shor, S.; Tal, O.; Maymon, R. [The second victim: treating the health care providers]. *Harefuah*. **2017***, 156,* 38–40.	Full-text not available
80	Sirriyeh, R. Coping with medical error: the case of the health professional. Available online: https://www.academia.edu/1723799/Coping_with_medical_error_the_case_of_the_health_professional	Mismatch with inclusion criteria
81	Stewart, K.; Lawton, R.; Harrison, R. Supporting "second victims" is a system-wide responsibility. *BMJ.* **2015**, *350*, h2341.	Mismatch with inclusion criteria
82	Stone M. Second victim support programs for healthcare organizations. *Nurs Manage*. **2020***, 51,* 38–45.	Full-text not available
83	Stone M. Second Victim Support: Nurses' Perspectives of Organizational Support After an Adverse Event. *J. Nurs. Adm.* **2020***, 50,* 521–525.	Mismatch with inclusion criteria
84	Stone, R.S.B. Code Lavender: A tool for staff support. *Nursing*. **2018***, 48,* 15–17.	Wrong focus
85	Strametz, R.; Raspe, M.; Ettl, B.; Huf, W.; Pitz, A. Handlungsempfehlung: Stärkung der Resilienz von Behandelnden und Umgang mit Second Victims im Rahmen der COVID-19-Pandemie zur Sicherung der Leistungsfähigkeit des Gesundheitswesens [Recommended actions: Reinforcing clinicians' resilience and supporting second victims during the COVID-19 pandemic to maintain capacity in the healthcare system]. Zentralbl. Arbeitsmed. Arbeitsschutz. Ergon. **2020**, doi: 10.1007/s40664-020-00405-7.	Wrong focus
86	Taifoori, L.; Valiee, S. Understanding or nurses' reactions to errors and using this understanding to improve patient safety. Ornac. J. **2015,** *33,* 13–22, 32–42.	Wrong focus
87	Tan, R.; Luo, K.; Hu, D.; Zhao, Y.; Han, Y.; Xu, K. Inpatient Suicide Second Victim Experience and Support Tool: Psychometric properties of a scale for nurses who experienced inpatient suicide at Chinese general hospitals. *Nurs. Health. Sci.* **2020***, 22,* 1111–1120.	Mismatch with inclusion criteria
88	Tanabe K, Janosy N, Vogeli J, Brainard A, Whitney G. Caring for the caregiver following an adverse event. Paediatr. Anaesth. **2021***, 31,* 61–67.	Mismatch with inclusion criteria
89	The AHSN Network. Second Victim Support Unit Scoping Project. Available online: https://www.ahsnnetwork.com/case-study/second-victim-support-unit	Wrong focus

90	Tobin, W.N. MITSSL Supporting patients and families for more than a decade. 2013. Available online: https://www.psqh.com/analysis/mitss-supporting-patients-and-families-for-more-than-a-decade/	Wrong focus
91	Vaithilingam, N.; Jain, S.; Davies, D. Helping the helpers: debriefing following an adverse incident. Obstet. Gynaecol. **2008**, *10*, 251–256.	Wrong focus
92	van Buschbach, S.; van der Meer, C.A.I.; Dijkman, L.; Olff, M.; Bakker, A. Web-Based Peer Support Education Program for Health Care Professionals. *Jt. Comm. J. Qual. Patient. Saf.* **2020**, *46*, 227–231.	Wrong focus
93	Van Gerven, E.; Seys, D.; Panella, M.; Sermeus, W.; Euwema, M.; Federico, F.; Kenney, L.; Vanhaecht, K. Involvement of health-care professionals in an adverse event: the role of management in supporting their workforce. *Pol. Arch. Med. Wewn*. **2014,** *124*, 313–320.	Wrong focus
94	Van Gerven, E.; Bruyneel, L.; Panella, M.; Euwema, M.; Sermeus, W.; Vanhaecht, K. Psychological impact and recovery after involvement in a patient safety incident: a repeated measures analysis. *BMJ. Open.* **2016**, *6*, e011403.	Wrong focus
95	van Steijn, M.E.; Scheepstra, K.W.F.; Yasar, G.; Olff, M.; de Vries, M.C.; van Pampus, M.G. Occupational well-being in pediatricians-a survey about work-related posttraumatic stress, depression, and anxiety. *Eur. J. Pediatr.* **2019**, *178*, 681–693.	Wrong focus
96	Vinson, A.E.; Randel, G. Peer support in anesthesia: turning war stories into wellness. *Curr. Opin. Anaesthesiol.* **2018**, *31*, 382–387.	Mismatch with inclusion criteria
97	Wahlberg, Å.; Högberg, U.; Emmelin, M. Left alone with the emotional surge - A qualitative study of midwives' and obstetricians' experiences of severe events on the labour ward. *Sex. Reprod. Healthc.* **2020**, *23*, 100483.	Mismatch with inclusion criteria
98	Washington Patient Safety Coalition (WPSC). Northwest Second Victim Programs. Foundaiton for Health Care Quality: Seattle, WA, USA, 2013.	Full-text not available
99	West, C.P.; Dyrbye, L.N.; Rabatin, J.T.; Call, T.G.; Davidson, J.H.; Multari, A.; Romanski, S.A.; Hellyer, J.M.; Sloan, J.A.; Shanafelt, T.D. Intervention to promote physician well-being, job satisfaction, and professionalism: a randomized clinical trial. *JAMA. Intern. Med.* **2014**, *174*, 527–533.	Wrong focus
100	White, A.A.; Brock, D.M.; McCotter, P.I.; Hofeldt, R.; Edrees, H.H.; Wu, A.W.; Shannon, S.; Gallagher, T.H. Risk managers' descriptions of programs to support second victims after adverse events. *J. Healthc. Risk. Manag*. **2015**, *34*, 30-40.	Wrong focus
101	White, A.; Waterman, A.; McCotter P.I.; Boyle, D.; Gallagher, T. Supporting health care workers after medical error: considerations for health care leaders. *J. Clin. Outcomes. Manag*. **2008**, *15*, 240–247.	Wrong focus
102	Whittle, S.N.; Horton-Deutsch, S. Reducing the Second Victim Phenomenon: Healing Our Healers With Caritas Coaching. *Holist. Nurs. Pract.* **2019,** *33*, 360–365.	Mismatch with inclusion criteria
103	Winning, A.M.; Merandi, J.; Rausch, J.R.; Liao, N.; Hoffman, J.M.; Burlison, J.D.; Gerhardt, C.A. Validation of the Second Victim Experience and Support Tool-Revised In the Neonatal Intensive Care Unit. *J. Patient. Saf.* **2020**, doi: 10.1097/PTS.0000000000000659.	Wrong focus

104	Zhang, X.; Chen, J.; Lee, S.Y. Psychometric Testing of the Chinese Version of Second Victim Experience and Support Tool. *J. Patient. Saf.* **2020**, doi: 10.1097/PTS.0000000000000674.	Wrong focus
105	No authors listed. Study Identifies Opportunities to Improve Support Programs for Clinicians Involved in Adverse Events. *Home. Healthc. Now.* **2016**, *34*, 7.	Mismatch with inclusion criteria
