# Supplementary material for: Promoting the Psychological Well-Being of Healthcare Providers Facing the Burden of Adverse Events: A Systematic Review of Second Victim Support Resources
Source: Int J Environ Res Public Health. 2021 May 11;18(10):5080. doi: 10.3390/ijerph18105080 (PMC8151650; doi:10.3390/ijerph18105080)
Supplement: Supplementary file 1 [file ijerph-18-05080-s001.zip › Supplementary File 4.pdf]

# SUPPLEMENTARY FILE 4. SUMMARY OF SECOND VICTIM SUPPORT RESOURCES AS DESCRIBED IN THE PRIMARY STUDIES

| **Support resource** | **Primary studies describing the support resource** | **Period and location of implementation** | **Conceptual basis** | **Short description of support resource** | **Outcomes reported in primary studies** | **Link to Website #** |
|---|---|---|---|---|---|---|
| Peer Support Service | Van Pelt, 2008 [37] | Inception in 2006 at Brigham and Women's Hospital (Boston, Massachusetts, USA) | - Model utilized by Kaiser Permanente under the leadership of Tony Devencenzi, Director of the Employee Assistance Program (EAP)<br>- Practices in Critical Incident Response and Psychological First Aid | - Peer support after adverse events<br><br>- Voluntary, confidential, immediately available<br><br>- One-on-one and group support<br><br>- If necessary, referral to higher levels of support<br><br>- Identification of affected staff: N/A<br><br>- Training required to become peer supporter | Pre-implementation survey:<br>Only 10% of healthcare providers who were offered or who found formal support services actually used them | N/A |
| Healing Beyond Today | Roesler et al., 2009 [34] | Implementation in September 2006 in Neonatal Intensive Care Unit at Riley Hospital for Children at Methodist Hospital Indianapolis (Indianapolis, USA) | - Just Culture model<br>- Critical Incident Stress Debriefing | - Support provided by trained outside facilitators four weeks after the event happened<br><br>- Attendance mandatory for unit staff<br><br>- One-on-one and group support<br><br>- If necessary, referrals to higher levels of support were possible<br><br>Identification of affected staff:<br>- Support offered to all providers involved in incident | Qualitative description:<br>- Debriefing and healing sessions, including grief exercises, essential for staff moving through the grief process<br>- Sessions as safe place to express pain and loss<br>- Six staff members out of seven returned to work, one followed her husband out of state | N/A |
| forYOU Team | - Scott et al., 2010 [35]<br>- Hirschinger et al., 2015 [27] | Implementation in March 2009 at University of Missouri Health Care (MU Health Care) (Columbia, Missouri, USA) | - Medically Induced Trauma Support Services (MITSS)<br>- Critical Incident Stress Management (CISM)<br>- Theory of Transpersonal Caring<br>- Scott Three-Tiered Interventional Model of Second Victim Support | - Peer support after a stressful clinical event<br><br>- Voluntary, confidential, immediately available<br><br>- One-on-one and group support<br><br>- If necessary, referral to higher levels of support<br><br>Identification of affected staff: | **Outcomes reported by Scott et al., 2010:**<br>Pre-implementation survey with healthcare staff:<br>- 30% experiencing distress because of a clinical patient safety event<br>- 35% of second victims received supports from colleagues/peers, 29% received support from supervisors<br>- 83% were for internal support, 6% for both internal/external support, 1% for external support; no preferences stated by 9%; according to < 1 % no support necessary<br>- Preferred format: support offered by the institution (at the department/unit level), immediately available, prompt, easy access to professionally trained counselors<br>- Support program as space to compose oneself after event | https://www.muhealth.org/about-us/quality-care-patient-safety/office-of-clinical-effectiveness/foryou |

| | | | | | | |
|---|---|---|---|---|---|---|
| | | | | - Identification by colleagues/peers and individual unit leaders<br>- Self-identification<br><br>- Training required to become a peer supporter<br><br>- Ongoing mentoring in regular meetings | **Outcomes reported by Hirschinger et al., 2015:**<br>In the first five years:<br>- Support for 1075 clinicians (mentoring: n=47 ("Tier 1"), group debriefing: n=632, one-on-one support: n=396 ("Tier 2"))<br>- Approximately one third required follow up ("Tier 3")<br><br>"Tier 1" (Department support and leadership monitoring):<br>- 62% (n=29) offered to nursing leaders<br>- Different reasons for mentoring interactions, e.g., unanticipated patient outcome (28%, n=13) not related to medical error (e.g., unexpected patient decline, fetal death, organ donation), personal or professional crisis (e.g., unexpected deaths/suicide of co-workers, workplace violence with clinician injury) (55%, n=26), adverse event (associated with medical errors) (17%, n=8)<br><br>"Tier 2" (Peer support encounters):<br>- 479 peer support encounters including 1,028 healthcare providers*****<br><br>- Group encounters: 83 sessions with 632 clinicians (17.3% of all peer support encounters )******<br>• Average group size: n=8<br>• Average encounter length: 1 h<br>• Reasons for group encounters: unforeseen/unanticipated patient outcomes (65%), personal/professional crisis experienced by an individual healthcare team member (33%), adverse event related to a medical error with multiple clinicians involved (2%)<br>• Most common risk factors evoking second victim response and requiring group support: complicated pediatric case, first death experiences, unexpected patient demise, death of a long-term patient, multiple patients with poor outcomes within a short period of time.<br><br>- One-on-one encounters: n=396 (82.7% of all peer support encounters)******<br>• Average encounter duration: 24 min<br>• Staff supported by discipline: nurses (53%), physicians (attending physicians, fellows, residents) (23%), unlicensed staff members (including students, volunteers, clerks, dietary, environmental services) (17%), other (including pharmacists, social workers, therapists, paramedics) (6.3%)<br>• Reasons for one-on-one activations: unanticipated patient outcome (55%), personal or professional crisis (28%), adverse event related to medical error (17%)<br>• one-on-one encounter preferred method after medical error<br>• Most common risk factors evoking second victim response and requiring one-on-one support: pediatric case, first death under "their watch", unexpected patient demise, long term patient, multiple patients with bad outcomes, patient known to staff member, patients that reminds staff of their family, young adult patient, organ donation community high profile patient<br><br>"Tier 3" (Professional resources, e.g., employee assistance program, personal counselor, chaplain, clinical health psychologist):<br>- Professional referrals required by 9.7% (n=104) individuals | |
| MITSS (Medically Induced Trauma Support Services) Tools: | Pratt et al. 2012 [33] | Publication of the toolkit in December 2010 on the Medically Induced Trauma Support Service (MITSS) website | Review of the literature and online documents by an expert panel | - Checklist of specific aspects to be addressed when developing and implementing an emotional support program for health care providers | In the first 12 months after publishing the toolkit:<br>- 6261 people visited the toolkit website<br>- 725 requested a download<br><br>Follow-up questionnaire with visitors of the website (n=36): | https://betsylehmancenterma.gov/initiatives/clinician-support?utm_source=mitss&utm_medi |

| | | | | | | |
|---|---|---|---|---|---|---|
| Tools for Building a Clinician and Staff Support Program | | | | - Guidance for healthcare organizations<br><br>- Identification of affected staff: N/A | - 75% rated the toolkit extremely/very helpful<br>- 88.9% found it easy to navigate<br>- 96.2% liked the format<br>- 62.5% used the toolkit to make positive changes in their institution | um=website&utm_campaign=redirect (original website not available) |
| Swaddle (Staff Well-being Assistance During Difficult Life Events) | - Trent et al., 2016 [36] | SWH Risk Management (RM) informal staff support (from 2001 to 2012)<br><br>→ formalized program: Swaddle (Staff Well-being Assistance During Difficult Life Events) (formalization started in 2011)<br><br>at Scott & White Healthcare (SWH) (Central Texas, USA) | - Previous experience with informal provider support<br>- Empirically supported crisis intervention training<br>- Medically Induced Trauma Support Services (MITSS) conference in Boston, Massachusetts in 2011<br>- MITTS program development toolkit<br>- Peer support methodologies<br>- Scott Three-Tiered Interventional Model of Second Victim Support<br>- Psychological First Aid | - Peer support for staff experiencing healthcare adversity<br><br>- Voluntary, confidential, immediately available<br><br>- One-on-one support<br><br>- If necessary, referral to higher levels of support<br><br>Identification of affected staff: N/A<br><br>- Training required to become peer supporter | Recommendations/Indications by 6 focus groups with 20 participants:<br>- Support by a peer important for impact, peer-to-peer support with a similar history of training/specialty is preferred<br>- Confidential and personalized support in protected environment important<br>- Incorporating of proactive education for health care professionals, particularly in legal processes, and the role of the provider into a support program<br>- Providing a timely response and support<br>- Communication necessary for coping with the event<br>- Importance of leadership communicating support for staff | N/A |
| RISE (Resilience in Stressful Events) | - Edrees et al., 2016 [25]<br>- Dukhanin et al., 2018 [24]<br>- Connors et al., 2021 [23] | Hospital-wide implementation between January 2010 and June 2012 at Johns Hopkins Hospital (Baltimore, Maryland, USA)<br><br>Pilot tested in 2011-2012 in the Department of Pediatrics at the Johns Hopkins Hospital (JHH) (Baltimore, Maryland, USA) | - local staff perceptions of second victim problem<br>- Medically Induced Trauma Support Services (MITSS) Toolkit<br>-for YOU Program at MU Health Care<br>- Psychological First Aid<br>- Social Resilience Model and G.R.A.C.E. ("Gathering attention, recalling intention, attuning, considering, engaging") <br>- self-evaluation from peer responders | - Peer support after stressful patient-related events, including adverse events, medical errors, deaths, unexpected outcomes, non-accidental trauma, difficult or violent interactions<br><br>- Voluntary, confidential, immediately available<br><br>One-on-one and group support<br><br>- If necessary, referral to higher levels of support<br><br>Identification of affected staff:<br>- Self-identification (paging the RISE team)<br>- Peer Identification (paging the RISE team)<br><br>- Training required to become a peer supporter, as well as attendance of monthly RISE team meetings and debriefings after encounters | **Outcomes reported by Edrees et al., 2016** (mixed-methods design)<br>- Peer responders (n=30 in 2016): registered nurses (63.3%), administration (10.0%), and others (e.g., staff from patient safety, child life, medicine) (26.7%)<br><br>Pre-implementation survey with healthcare staff (n=144):<br>- Multidisciplinary peer group preferred by 68.7% of respondents, a nurse manager by 15.5%, pastoral care by 13.3%<br>- Individual support preferred by 70.7% of respondents<br>- Preferred support immediately after event (12.7%, n=17), a few hours after event (25.4%, n=34), a couple of days after the event (48.2%, n=66), a week after the event (8.1%m n=11), or depending on the severity of the event/comfort level in the aftermath<br><br>First 52 months of program:<br>- 119 calls including ca. 500 individuals (one-on-one encounters: 43%, n=34, group sessions: 56%, n=44, unknown: 2.5%, n=2, with an average interaction of 49 minutes)<br>- Increase in calls throughout this time from ca. 1 to 4 calls/month<br>- Callers by professions (for 80 encounters): nurses 56.3%, multidisciplinary group 28.8%, physician 16.3%, nurse practitioners 3.8%, other (e.g., nurses' aide, respiratory technician) 6.3%, not recorded 13.8%<br>-Incidents related to death of a patient (45%), adverse events (21.3%), medical errors (only 5%), or other situations (e.g., difficult decisions, burnout, staff assault, intrastaff conflicts)<br>- Callers looking for support at recommendation of their supervisors (56.2%), self-referred (21.3%), peers (11.3%), nurse leaders (5%), unknown (8.8%)<br>- Experience of barriers with accessing RISE reported by 8% of callers (primary barrier: lack of awareness on how to access the program)<br><br>Survey with RISE peer supporters<br>- on RISE training: | https://www.johnshopkinssolutions.com/solution/rise-peer-support-for-caregivers-in-distress/ |

| | | | | | | |
|---|---|---|---|---|---|---|
| | | | | | - higher comfort levels in responding to the second victim after training compared to their rating of their overall competence in meeting the callers' needs (excellent/very good 44.9% of the time)<br>- RISE training considered as enriching, in particular, role-play focusing on key principles of peer response seen as very effective method<br>- on the peer encounters:<br>- Additional support sources offered by 84.3%<br>- Success of interaction rated as excellent or neutral by 66.7% and 22.8% of peer responders, respectively<br>- Little or no emotional stress after peer encounter indicated by ca. 70% of peer responders<br>- 87.8% of peer supporters confident to have met second victims' expectations<br>- 82.4% of peer supporters satisfied with the interaction<br><br>Focus Groups with RISE peer supporters (n=5):<br>- Initial psychological first aid and ongoing training (peer responder meetings, debriefings) considered as helpful<br>- Higher levels of personal distress during group than one-on-one encounters<br>- Need for more training on group sessions with multiple professions, training focused on different professions (physicians and other professions) and the development of key phrases and scripts for the beginning and the end of an encounter.<br><br>**Outcomes reported by Dukhanin et al., 2018** (mixed-methods design, i.e., quantitative pre-implementation and four-year follow-up survey; open ended questions; sample: healthcare staff in Department of Pediatrics):<br>- Similar levels of willingness to contact organizational support (58% vs. 60%) and familiarity with term second victim (56% vs. 65%, p=.053) at baseline and follow-up<br>- At follow up, lower levels of desirability to speak to someone regarding an unanticipated clinical event than pre-implementation (85% vs. 71%, p=.002)<br>- At follow-up, greater perception of availability (41% vs. 60%) and benefits of support (85% vs. 94%) (p<.001 and p=.014, respectively)<br>- 93% of the respondents who had used RISE or knew someone who had used it very likely to recommend RISE to others (100% of nurses, 73% of physicians)<br><br>Content analysis:<br>- Various preferences for form of support and specific interventions (in particular, anonymity, non-judgmental approach, 24/7 access, commitment to follow-up, active listening, demonstrating compassion, validation/debriefing of feelings)<br>- Personal experience with RISE stated by 20 respondents: RISE seen as useful by majority, barriers, such as overcoming blame culture, need to promote the initative, need for more time to handle adverse events mentioned by few respondents.<br><br>**Outcomes reported by Connors et al., 202**1 (Survey with RISE peer supporters):<br>Between 2012 and 2018:<br>- 59 persons joined RISE (all completed training and participated in at least one encounter)<br>- RISE annual turnover rates: 0% (2012), 14% (2013), 22% (2014), 26% (2015), 0% (2016), 9% (2017), and 14% (2018), with a mean annual turnover rate of 12% (leaving RISE but remaining at same job position, leaving and changing job position within JHH, leaving JHH)<br>- 19 out of 26 respondents were RISE members for 3 years or more<br>- 8 out of 25 members participated in more than 10 RISE encounters | |

| | | | | | | |
|---|---|---|---|---|---|---|
| | | | | | - Positive/strongly positive view about personal autonomy in performing RISE duties reported by 84% of RISE members<br>- Positive/strongly positive view about meaningfulness of RISE duties reported by 100% of RISE members<br>- 96% of RISE members satisfied/strongly satisfied with RISE duties<br>- Positive/strongly positive perception of the impact of RISE reported by 93% of RISE members<br>- 89% of RISE members positive/strongly positive in assessing their level of RISE competencies<br>- 72% of RISE members disagreed/strongly disagreed that RISE duties were cause for burnout<br>- Positive/strongly positive perception of personal resilience reported by 56% of RISE members<br>- Confidence in individual RISE competencies indicated as strongly positive (28-76% across competencies), positive (20-60%), neutral (4%-12%), no negative self-assessment<br>- Strong significant association between perceived level of resilience and tenure with RISE ($p<.001$)<br>- No statistically significant associations between perceived level of autonomy in RISE duties and tenure with RISE, between personal resilience and number of RISE encounters, between perceived level of burnout resulting from RISE duties and tenure with RISE, and between level of burnout and number of RISE encounters<br><br>Content analysis:<br>positive perceptions of RISE volunteering, personal empowerment thanks to RISE, personal affinity with RISE and increased energy and enjoyment from RISE membership reported by participants | |
| Care for the Caregiver Program | Morales & Brown, 2019 [32] | Implementation ca. 2013 in a Ten-Hospital Health System (District of Columbia, USA) | - Communication and optimal resolution (CANDOR) toolkit<br>- National Transportation Safety Board Go Team<br>- Scott Three-Tiered Interventional Model of Second Victim Support<br>-Immediate emotional first aid | - Peer support after serious, unanticipated adverse events<br><br>- Expansion to other events (e.g., divorce, loss of a loved one, burnout, domestic abuse)<br><br>- Voluntary, confidential, immediately available<br><br>- One-on-one support<br><br>- If necessary, referral to higher levels of support<br><br>-Identification of affected staff:<br>• Identification by colleagues or patient safety managers<br>• Self-identification<br><br>- Training required to become peer supporter | N/A | N/A |

| | | | | | | |
|---|---|---|---|---|---|---|
| YOU Matter Program | - Krzan et al., 2015 [28]<br>- Merandi et al., 2017 [30] | Implementation from July 2013 to March 2014 at the Department of Pharmacy, Nationwide Children's Hospital (Columbus, Ohio, USA) | - Scott Three-Tiered Interventional Model of Second Victim Support [Scott et al., 2010]<br>- MU Health Care's forYOU Team | - Peer support after medical errors or adverse patient outcomes<br><br>- Voluntary, confidential, immediately available<br><br>- One-on-one and group support<br><br>- If necessary, referral to higher levels of support<br><br>Identification of affected staff:<br>- Self-identification through email to YOUMatter Program, YOUMatter hotline, hospital intranet<br>- Peer Identification<br><br>- Training required to become peer supporter | **Outcomes reported by Krzan et al., 2015**:<br>Pre-implementation survey with healthcare staff:<br>- 30% (36/120) of staff involved in second victim events<br>- 25% (30/121) of staff received support from a member of the pharmacy department after an adverse event<br>- 93.3% (113/121) saw the need for a program<br><br>Postimplementation survey with healthcare staff:<br>- Personal use of YOU Matter program by 2.5% (3/121)<br>- Referral of a coworker for peer support through YOU Matter program by 11.0% (11/121)<br>- Program beneficial for pharmacy department indicated by 85% (95/121)<br>- 3 individuals reported in free-text response that they benefited from the experience.<br><br>**Outcomes reported by Merandi et al., 2017:**<br>- 300 peer supporters trained, 30 leaders identified<br>- Peer supporters by discipline: nurses (44%), physician (6%), non-clinical staff (5%), respiratory therapy (5%), pharmacists (4%), social workers (3%), nurse practitioner (3%), other staff (multiple disciplines including clinical and nonclinical staff) (30%)<br>- Since program inception 253 encounters (i.e., 232 peer and 21 group encounters)*****<br>- Peer encounters by location: emergency department (62%), ICU (8%), pharmacy (7%), operating room/surgery (4%), homecare (1%), other (18%)<br>- Second victims by discipline******: nurses (32.32%), patient care assistants (14.2%), physicians (8.2 %), social workers (8.2%), pharmacists (7.8 %), medics (6.0 %), unit clerks (3.4 %), respiratory therapists (2.6 %), security (2.6 %), other staff (e.g., nurse anesthetists, interpreters) (14.6 %)<br>- Six common reasons for peer encounters: patient death, emotional stress, cardiac arrest, medication error, and alleged child abuse | https://www.nationwidechildrens.org/careers/you-matter-program |
| Washington University School of Medicine (WUSM) Peer Support Program | Lane et al., 2018 [29] | Implementation in April 2014 at the Barnes-Jewish Hospital (St. Louis, Missouri, USA); | N/A | - Peer support after medical errors and adverse events<br><br>- Voluntary, confidential, available near the time of a serious event<br><br>- One-on-one support<br><br>- If necessary, referral to higher levels of support<br><br>Identification of affected staff:<br>- Identification by safety/risk management staff and peer support provider<br>- Self-identification<br>- Pro-active contacting of clinicians involved in serious medical errors/adverse events<br><br>- Training required to become peer supporter | Data collection between April 2014 and January 2017:<br>- 88 clinicians nominated as supporter by department chairs or through self-nomination<br>- 36 clinicians included in clinician peer supporter pool (including 44.4% (n=16) of clinicians from surgical or procedural specialties)<br>-Use of the program by 165 individuals (41.2% residents, 10.3% fellows, 42.4% faculty members, 3.6% nurse practitioners/physician assistants, 2.4% certified registered nurse anesthetists.<br>- 4.8% individuals on average referred per month (range: 0-12)<br>- Follow-up declined by 10.3% (n=17)<br>- Median of two interactions (range: 1-10)<br>- Referral to higher level of support required by 9.7% of supported staff | https://collaboration.wustl.edu/depts/patientsafety/supportforclinicians/Pages/WUSMClinicianSupport.aspx |
| Second victim support | Wijaya et al., 2018 [38] | Study conducted (treatment given) from 2015 to 2017 at | forYOU 2010 toolkits | - Peer support after adverse events | - Significant increase in patient safety culture (measured with the HSOPSQ) Hospital Survey on Patient Safety Culture (HSOPSC) among healthcare staff in | N/A |

| | | | | | | |
|---|---|---|---|---|---|---|
| program (no specific name) | | the Bali International Medical Centre (BIMC) Hospitals Kuta and Nusa Dua (Bali, Indonesia) | | - One-on-one and group support<br><br>- Identification of affected staff: N/A | both hospitals after the implementation of second victim support program (p=.007) (i.e., between first and second phase)<br>- No significant difference in patient safety culture between second and third phase (one year after the second phase) | |
| MISE (Mitigating Impact in Second Victims) | Mira et al., 2017 [31] | Evaluation of MISE between November 2015 and February 2017 at the San Juan de Alicante Hospital (Alicante, Spain) | Based on patient safety and, in particular, second victim literature | - Program provides information to professionals about the second victim phenomenon in nine weeks of online training<br><br>- Users can individually search the website for information<br><br>- Identification of affected staff: N/A | - Advanced Accreditation for health websites after fulfilling the requirements<br>- Comprehension and practical value of MISE positively assessed by 88% and 92% of patient safety manager, respectively (sample size: n=26)<br>- Positive evaluation of MISE by healthcare professionals (sample size: n=266) (8.8 points out of 10)<br>- Increase of knowledge on patient safety terminology, prevalence and impact of adverse events/errors, second victim support models, and recommended actions in the aftermath of severe adverse events (p <.001) (sample size: n= 266) | http://www.segundasvictimas.es |
| Surgery-specific Second Victim Support Program (no specific name) | El Hechi et al., 2019 [26] | Implementation in 2017 at the Department of Surgery at Massachusetts General Hospital (Boston, Massachusetts, USA) | - Systematic literature review on the topic<br>- Scott Three-Tiered Interventional Model of Second Victim Support<br>- Series of multidisciplinary expert meetings<br>- Adaption from PTS at the Brigham and Women's Hospital | -Peer support after major perioperative adverse events, including intraoperative adverse events and catastrophic and/or unexpected postoperative outcomes<br><br>- Voluntary, confidential, outreach to affected staff in the aftermath of the event<br><br>- One-on-one support<br><br>- If necessary, referral to higher levels of support<br><br>Identification of affected staff:<br>- Use of multifaceted approach to identify major perioperative adverse events<br><br>- Training required to become peer supporter | - Within 1 year: 47 outreach interventions (similar numbers for attendings and trainees)<br>- 19% of affected peers did not want peer support<br>- Every peer supporter participated at least in one intervention<br>- Majority of adverse events: intraoperative mishaps, unexpected patient deaths shortly after surgical intervention<br>- Outreach to surgeons from all included surgical specialties and to surgical trainees at all levels of training<br><br>- Communication between peer support and affected peers: face-to-face meetings (52%), email (45%), phone calls (32%)<br>- Participants satisfied with program's confidentiality (89%), its safe/trusting environment (73%), timeliness of intervention (83%)<br>- Positive impact of the program on the department's safety and support culture indicated by 81% of participants<br>- 2 referrals to the employee assistance program, 1 referral to the ED for a psychiatric assessment (6.4% of peer support encounters)**<br>- Areas for improvement, including process for identifying events requiring outreach, increasing departmental awareness of the program, providing opportunity for affected individuals even when an adverse event was not identified<br>- Most free-text comments from supporters and affected peers positive | N/A |

Notes:
***** We summed up the absolute frequencies reported for group and one-on-one encounters.
****** We calculated the respective percentage.
# Except for Mira et al. [ref] and Pratt et al. [ref], the links to the websites were not mentioned in the included studies but searched by us.

Abbreviations:
HSOPSC: Hospital Survey on Patient Safety Culture; N/A: Not available
